# A novel method for evaluating load restraint assemblies to ensure the safety of railway freight transportation

**DOI:** 10.1038/s41598-024-54772-9

**Published:** 2024-02-26

**Authors:** Duo Zhang, Su-Mei Wang, Yin-Ying Tang, Yi-Qing Ni, Jing-Wei Guo, Qi-Yuan Peng

**Affiliations:** 1 School of Transportation and Logistics, Southwest Jiaotong University, Chengdu, People’s Republic of China; 2 National Rail Transit Electrification and Automation Engineering Technology Research Center (Hong Kong Branch), Hong Kong, People’s Republic of China; 3 Department of Civil and Environmental Engineering, The Hong Kong Polytechnic University, Hong Kong, People’s Republic of China; 4 National United Engineering Laboratory of Integrated and Intelligent Transportation, Southwest Jiaotong University, Chengdu, People’s Republic of China; 5 Faculty of Business, City University of Macau, Macau, People’s Republic of China

**Keywords:** Vehicle running safety, Goods vibration, Lashing stiffness, Multibody dynamics simulation, Railway freight transportation, Mechanical engineering, Civil engineering

## Abstract

The violent goods vibration during curve negotiation is a huge threat to the vehicle running safety. Qualified load restraint assemblies that can significantly suppress the cargo vibration are necessary. This study proposes a novel method for evaluating the essential restraint strength, focusing on the relative motion between cargo and wagon. In the beginning, as a comparison, current methods are used to calculate the necessary stiffness of lashings, which are adopted to restrain the cargo vibration on the wagon. Based on the data of the field test, the accuracy of the established wagon-cargo coupled dynamics model is validated. The loaded wagon model negotiates the curve under different running and loading conditions. The simulation results and analysis demonstrate effective strategies for suppressing the vibration of the cargo and reveal the necessary lashing stiffness. The comparison among the results of different evaluation methods shows that the stability of the cargo can be improved by optimizing the lashing stiffness with the method of dynamics simulations. We hope this study will make a positive contribution to the safety of railway freight transportation.

## Introduction

**Figure 1:**
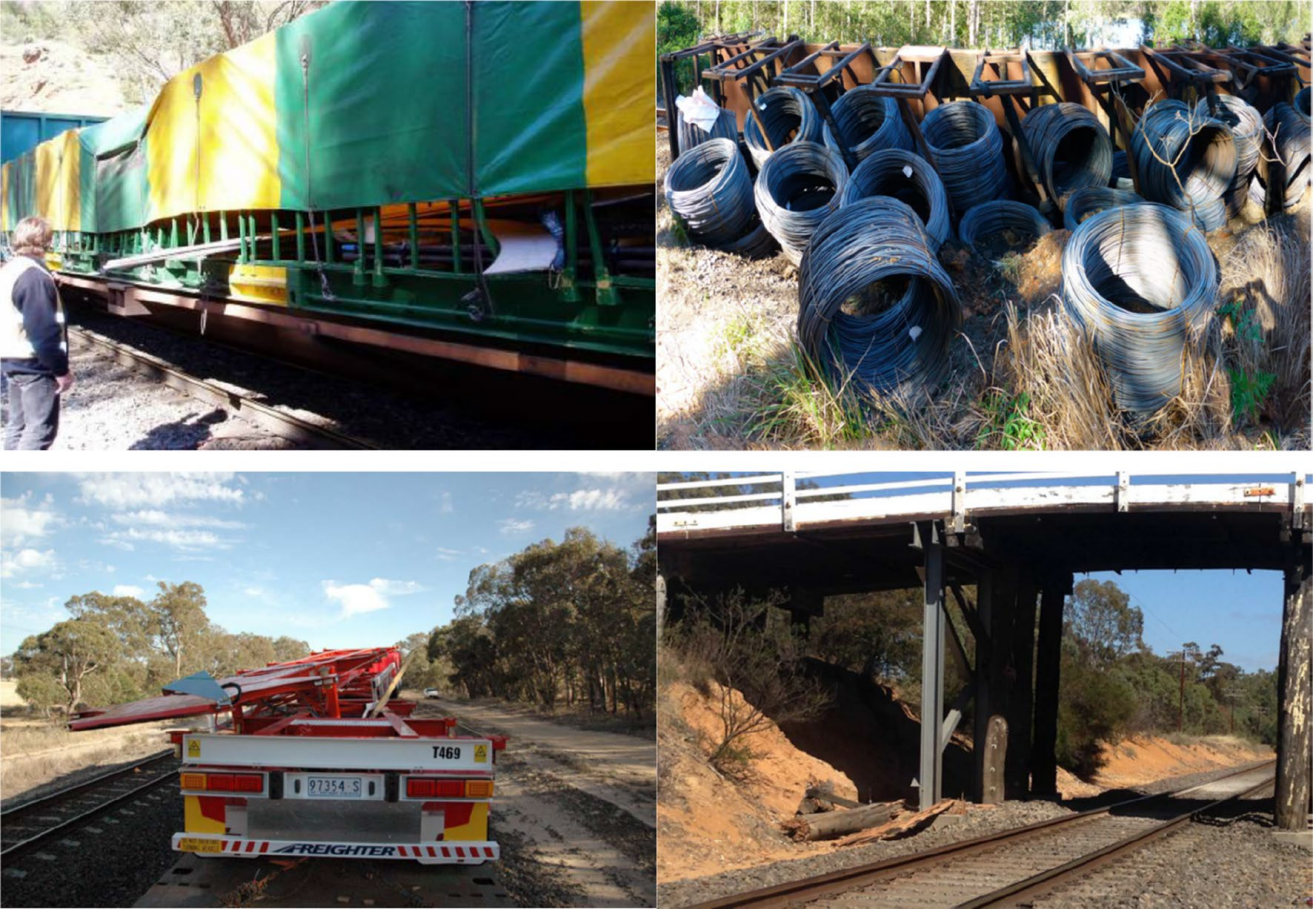
The consequence of the shift of goods^2–4^.

The railway plays a critical role in freight transportation, especially for the long-distance transportation. For cargoes which are not fixed connected with the carbody of freight wagon, the relative movement between the cargo and carbody is unavoidable and will aggravate the carbody vibration and cargo damage. In order to prevent the train accident, the violent vibration and shift of goods must be constrained^1^. Thus, load restraint assemblies, such as the wire ropes or the web lashing made from man-made fibers, are requisite. Nevertheless, the deficiency of securing forces used to result in the shift of the goods so as to play an important role in the occurrence of train accidents and subsequent disruption to the environment, as is shown in Fig. 1.

Even though different rules covering the evaluation of necessary securing forces have been published by many organizations^5–8^, there is a common limitation in all the present methodologies. These rules focus on the accelerations of goods in the global coordinate system and make them the basis for calculating securing forces. The vibration of goods relative to the wagon, which is a critical threat to the safety of railway freight transportation, is ignored in the current rules.

Obviously, the calculation of securing forces should consider the stability of goods both laterally and longitudinally. Some scholars paid attention to the longitudinal acceleration of goods. They considered the vibration of goods on the wagon during braking and shunting. The longitudinal acceleration of goods was proved to be less than 5 m/s^2^ when the shunting speed was smaller than 4 km/h^9^. Different mathematical models were established to investigate the lading longitudinal acceleration^10–12^. Although these studies did not measure the longitudinal movement of goods relative to the wagon, at least the goods were investigated separately from the wagon.

By contrast, the lateral and vertical accelerations of goods, which increase significantly during curve negotiations, deserve more attention. Most scholars focused on the stability of the wagon^13–19^. The investigations about the lateral and vertical vibrations of goods are very limited. The relationship between the lateral vibration of the cargo and the lashing stiffness was studied^20^, but it was assumed that the cargo was loaded symmetrically and the difference between the processes of negotiating the circular curve and the transition curve was ignored. Aiming at the deficiencies of former studies, we implement the research on the lateral stability of goods.

Due to the wide applicability, we take the diagonal lashings as the load restraint assemblies in this paper. We focus on the lateral and vertical vibrations of goods, and therefore investigate the process of curve negotiations for the railway wagon.

The remainder of this paper is organized as follows: In Section “Methodology”, we calculate the necessary lashing forces with two representative methods respectively as a comparison. The multibody dynamics functions of the cargo are established and then we build the wagon-cargo coupled dynamics model. Section “Numerical verification” verifies the accuracy of the proposed dynamics model based on the field test data. In Section “Simulation results”, according to the simulation results, the influencing factors of railway freight transportation safety are discussed, and then the required lashing force can be confirmed. In Section “Discussion”, the contrast among the results of different evaluation methods demonstrates that the stability of the cargo can be significantly improved by means of the method of dynamics simulations. The conclusions of this paper are drawn in Section “Conclusions”.

## Methodology

### Calculation results by the current evaluation method

Many methodologies have been proposed by different organizations to calculate the necessary lashing force^5–8^. Some organizations, such as Chinese Railways (CR), suggest that the calculation results should be related to the cargo loading position. The other organizations, such as the European Committee for Standardization (CEN), define the calculation method despite the longitudinal offset of the wagon. In this paper, we focus on transportation safety during the curve negotiation. Thus, we merely discuss the load restraint assemblies from the perspective of lateral cargo stability.

**Figure 2:**
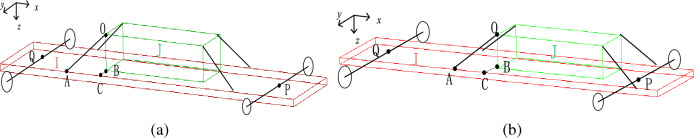
Schematic diagram of the loaded wagon: (**a**) Even-loaded; (**b**) Maximum longitudinal offset.

In this paper, we take the open-top wagon of C_70H_ as an example and we assume that the cargo is a homogeneous cube to simplify the problem. Four identical diagonal lashings are adopted as the load restraint assemblies. Then the cargo can be restrained as is shown in Fig. 2, where I denotes the floor of the wagon, J denotes the cargo, P and Q denote the center pivots of the front bogie and rear bogie respectively, O denotes one of the attachment points, A denotes one of the lashing points, B denotes the projection of O and C denotes the projection of B. Generally, the cargo is loaded evenly in the lateral direction. Thus, the wagon with the maximum longitudinal offset is investigated in this paper, as well as the even-loaded wagon.

The masses of the given cargo and the empty wagon are 50 tons and 23.8 tons respectively. The maximum longitudinal distance between the gravity centers of the cargo and the wagon, which means the longitudinal offset in this paper, can be calculated as 1.8 m according to their masses and the regulation of CR^6^. The value of |AC| is assumed as a constant of 2 m. As the computation basis, the values of primary parameters are listed in Table 1.

**Table 1:** Key parameters for the decision of load restraint assemblies.

Definition	Parameter	Value
Length of the wagon floor	*L*	13 m
Width of the wagon floor	*W*	2.892 m
Length of the cargo	*l*	4.3 m
Width of the cargo	*w*	1.5 m
Height of the cargo	*h*	1 m
Distance between center pivots	*d*	9.21 m
Longitudinal offset	*a*	1.8 m
Distance between A and C	|AC|	2 m
Distance between B and C	|BC|	0.696 m
Distance between B and O	|BO|	1 m
Distance between A and O	|AO|	2.34 m
Mass of the empty wagon	*M*	23.8 t
Mass of the cargo	*m*	50 t
Static friction coefficient on the contact surface between the cargo and wagon floor	*μ*_*s*_	0.51
Dynamic friction coefficient on the contact surface between the cargo and wagon floor	*μ*_*d*_	0.44

In Table 1, |AO| represents the length of the lashings. Then, based on the material mechanics and the test data, CR proposes the stiffness and lashing capacity for a certain type of wire rope in Table 2^6,21^, where *ϕ* (mm) denotes the diameter of the lashing, *k* (N/m) denotes the stiffness of the lashing and *LC* (kN) denotes the lashing capacity of the lashing.

**Table 2:** Characteristics of the lashings.

*ϕ*	6	7	7.7	8	9	9.3	10	11	12	12.5	13
*k*	1.69 × 10^6^	2.30 × 10^6^	2.79 × 10^6^	3.01 × 10^6^	3.81 × 10^6^	4.06 × 10^6^	4.70 × 10^6^	5.69 × 10^6^	6.77 × 10^6^	7.34 × 10^6^	7.94 × 10^6^
*LC*	9.25	12.55	15.85	16.4	20.8	22.8	25.65	31	36.9	40.52	43.3
*ϕ*	14	15.5	16	17	18	18.5	20	22	24	26	28
*k*	9.21 × 10^6^	1.13 × 10^7^	1.20 × 10^7^	1.36 × 10^7^	1.52 × 10^7^	1.61 × 10^7^	1.88 × 10^7^	2.27 × 10^7^	2.71 × 10^7^	3.18 × 10^7^	3.68 × 10^7^
*LC*	50	63.3	65.5	76.63	83	91.18	102.5	124	147.5	173	201

#### The method defined by CR

Step 1: Based on the field test and practical experience, the maximum inertial accelerations of the cargo are defined, and then the forces on the cargo during railway transportation are calculated.

1. The maximum lateral inertial force can be obtained as
  1 $$ N = n_{0} \times m\left( {{\text{kN}}} \right) $$
  where *n*_0_ denotes the maximum lateral acceleration of the cargo and
  2 $$ n_{0} = 2.82 + 2.2 \times \frac{a}{d}\left( {{\text{kN}}/{\text{t}}} \right) $$
2. The maximum vertical inertial force can be obtained as
  3 $$ U = u_{0} \times m\left( {{\text{kN}}} \right) $$
  where *u*_0_ denotes the maximum vertical inertial acceleration of the cargo and
  4 $$ u_{0} = 3.54 + 3.78 \times \frac{a}{d}\left( {{\text{kN}}/{\text{t}}} \right) $$
3. The minimum lateral friction force on the cargo can be obtained as
  5 $$ f = \mu ({9}.{8}m - U) \, \left( {{\text{kN}}} \right) $$
  where *μ* denotes the friction coefficient and is deemed as *μ*_*d*_ for CR.

Step 2: The stability of the cargo is evaluated to judge if the load restraint assemblies are necessary. First of all, it is important to decide if we should take extra measures to prevent the cargo from tilting over. Then, we need to judge the necessity of load restraint assemblies considering balancing the lateral inertial force of the cargo.

1. The lateral rolling coefficient of the cargo can be obtained as
  6 $$ \eta { = }\frac{9.8m\alpha }{{N\beta }} $$
  where *α* and *β* respectively denote the lateral and vertical distance between the gravity center of the cargo to the point of B.
  If *η* > 1.25, we can conclude that the cargo is impossible to tilt over during railway transportation.
2. The necessary tension force in the lateral direction is calculated as
  7 $$ \Delta N = 1.25N - f\left( {{\text{kN}}} \right) $$

Step 3: For the case whose $\Delta N$ is positive, the necessary restraint strength can be calculated. The minimum allowable stress of each lashing is given by

8 $$ S{ = }\frac{\Delta N|AO|}{{2|BC|}}\left( {{\text{kN}}} \right) $$

Based on the values illustrated in Table 1, it is easy to find out that the cargo will not tilt over and *S* equals 65 kN when the cargo is loaded as Fig. 2a shows. Likewise, for the cargo which is loaded as Fig. 2b illustrates, *S* equals 136 kN and there is no need to keep the cargo from tilting over. Then according to the data listed in Table 2, we can decide that the wire ropes of *ϕ*16 and *ϕ* 24 should be adopted as the lashing devices respectively.

#### The method defined by CEN

Compared with CR, CEN define a method which has a similar procedure and its expressions are more succinct.

Step 1: Inertial forces on the cargo are calculated.

1. The maximum lateral inertial force on the cargo can be obtained as Eq. (1) and CEN directly defines *n*_0_ as 0.5g.
2. The minimum difference between the vertical inertial force on the cargo and its gravity can be obtained as
  9 $$ F_{z} = c_{z} {\text{g}} \times m\left( {{\text{kN}}} \right) $$
  where *c*_*z*_ equals 1 when considering the possibility of tilting over and equals 0.7 when calculating the tension force for preventing the cargo from sliding.

Step 2: The possibility of tilting over for the cargo is assessed.

CEN proposes that it is not necessary to prevent the cargo from tilting over if the following inequality can be proved:

10 $$ \alpha > \frac{{n_{0} }}{{c_{z} g}} \times \beta $$

Thus, it is obvious that the selected cargo will not tilt over during railway transportation.

Step 3: The necessary tension force for each lashing device can be calculated as

11 $$ S = m \times g\frac{{\frac{{n_{0} }}{g} - \mu \times f_{\mu } \times c_{z} }}{{2(\cos \angle OAB \times \cos \angle ABC + \mu \times f_{\mu } \times \sin \angle OAB)}} $$

where $\mu$ denotes the friction coefficient which can be calculated as $(0.925 \times \mu_{s} + \frac{{\mu_{d} }}{0.925})/2$, *f*_μ_ denotes the conversion factor which is proposed as 0.75 by CEN.

Based on Eq. (11) and the values shown in Table 1, *S* equals 139 kN and the wire rope of *ϕ*24 should be adopted, whether the cargo is loaded evenly or not.

The comparison between the calculation results of the methods of CR and CEN reveals that these two methods are almost identical considering the worst loading position, and the method of CR is more specific because the factor of longitudinal offset is taken into account.

### Multibody dynamics model

In order to validate the effectiveness of the current evaluation methods, we establish the wagon-cargo coupled model, which is made up of one cargo, one carbody, and two three-piece bogies. Since the dynamics equations of the carbody and bogies have been proposed^22,23^, the dynamics function of the cargo plays an important role in building the wagon-cargo coupled model.

#### Multibody dynamics functions of the cargo

**Figure 3:**
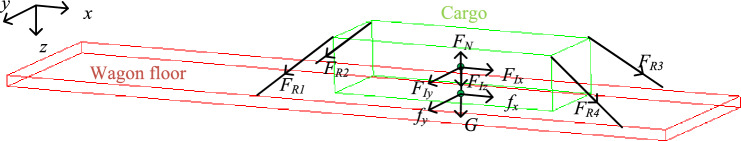
Forces on the cargo during transportation.

During railway transportation, the cargo is under the action of gravity, inertial forces, lashing forces and the contact force from the wagon floor, as is illustrated in Fig. 3, where *F*_*Ri*_ (*i* = 1, 2, 3, 4) denotes the lashing forces, *G* denotes the gravity of the cargo, F_*Ij*_ (*j* = *x, y, z*) denotes the inertial force of the cargo in each direction, *F*_*N*_ denotes the supporting force and* f*_*k*_ (*k* = *x, y*) denotes the friction forces on the contact surface. The four lashing devices are assumed as identical.

If lashing stiffness is sufficient, it can be assumed that the cargo keeps in complete contact with the wagon floor. Then the supporting force acts in the middle of the cargo, and the cargo may slide on the wagon floor or rotate around the axes through its gravity center. If we use *F*_*Ri*_^*x*^, *F*_*Ri*_^*y*^ and *F*_*Ri*_^*z*^ to represent the component forces of *F*_*Ri*_ in the corresponding direction, the dynamics function of the cargo can be expressed as

12 $$ \left\{ {\begin{array}{*{20}l} {m\ddot{X} = \mathop \sum \limits_{i = 3,4} F_{Ri}^{x} - \mathop \sum \limits_{i = 1,2} F_{Ri}^{x} + F_{Ix} + f_{x} } \hfill \\ {m\ddot{Y} = \mathop \sum \limits_{i = 1,4} F_{Ri}^{y} - \mathop \sum \limits_{i = 2,3} F_{Ri}^{y} + F_{Iy} + f_{y} } \hfill \\ {m\ddot{Z} = \mathop \sum \limits_{i = 1}^{4} F_{Ri}^{z} + F_{Iz} + G - F_{N} } \hfill \\ {J_{x} \ddot{\theta } = \mathop \sum \limits_{i = 1,4} \left( {F_{Ri}^{y} \cdot \frac{h}{2}} \right) - \mathop \sum \limits_{i = 2,3} \left( {F_{Ri}^{y} \cdot \frac{h}{2}} \right) + \mathop \sum \limits_{i = 1,4} \left( {F_{Ri}^{z} \cdot \frac{w}{2}} \right) - \mathop \sum \limits_{i = 2,3} \left( {F_{Ri}^{z} \cdot \frac{w}{2}} \right) - f_{y} \cdot \frac{h}{2}} \hfill \\ {J_{y} \ddot{\phi } = \mathop \sum \limits_{i = 1,2} \left( {F_{Ri}^{x} \cdot \frac{h}{2}} \right) - \mathop \sum \limits_{i = 3,4} \left( {F_{Ri}^{x} \cdot \frac{h}{2}} \right) + \mathop \sum \limits_{i = 1,2} \left( {F_{Ri}^{z} \cdot \frac{l}{2}} \right) - \mathop \sum \limits_{i = 1,4} \left( {F_{Ri}^{z} \cdot \frac{l}{2}} \right) + f_{x} \cdot \frac{h}{2}} \hfill \\ {J_{z} \ddot{\psi } = \mathop \sum \limits_{i = 1,3} \left( {F_{Ri}^{x} \cdot \frac{w}{2}} \right) - \mathop \sum \limits_{i = 2,4} \left( {F_{Ri}^{x} \cdot \frac{w}{2}} \right) + \mathop \sum \limits_{i = 2,4} \left( {F_{Ri}^{y} \cdot \frac{l}{2}} \right) - \mathop \sum \limits_{i = 1,3} \left( {F_{Ri}^{y} \cdot \frac{l}{2}} \right)} \hfill \\ \end{array} } \right. $$

where *X*, *Y* and *Z* denote the displacements of the cargo; *J*_*x*_, *J*_*y*_, and *J*_*z*_ denote the moments of inertia; *θ* denotes the roll angle; *ϕ* denotes the pitch angle; *ψ* denotes the yaw angle.

**Figure 4:**
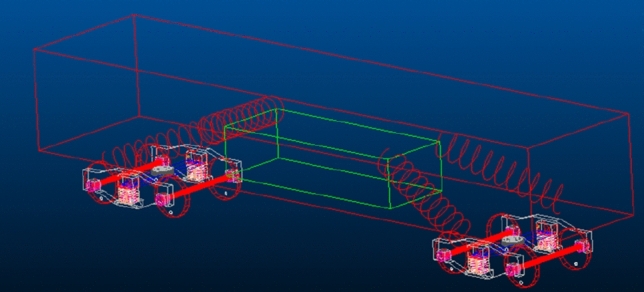
Multibody dynamics simulation model of the loaded wagon.

Subsequently, the wagon-cargo coupled model can be established by VI-Rail, which is one of the most popular multibody dynamics simulation platforms for trains^24–26^. The wheelset is connected to the axle box by the rubber pad. The side frame lies on the axle box and bears the suspension system. The load of the wagon is distributed to each bolster, and then transferred to the suspensions through the wedges. The contact force and lashing forces act between the cargo and the wagon. Figure 4 shows the simulation model and its key characteristics have been introduced in^27^.

#### Assessment indices

In order to verify the accuracy of the wagon-coupled model, the derailment coefficient (DE) and wheel unloading rate (UN) are tested during the simulations. The primary objective of this research is to depress the vibration of the cargo during the curve negotiation. Thus, we focus on the indices which can assess the lateral stability of the cargo, as well as the wagon.

First of all, the lateral and vertical accelerations of the cargo (represented by LA_c_ and VA_c_ respectively) in the reference system of the wagon floor should be collected. Moreover, the rolling acceleration of the cargo (RA) also needs to be considered. All these accelerations are measured from the gravity center of the wagon floor to the gravity center of the cargo.

The lateral and vertical accelerations of the wagon (represented by LA_w_ and VA_w_ respectively) are assessed to demonstrate the effect of the cargo stability on the wagon stability. Two sensors are set upon the center pivots of the trucks. The larger value which is monitored by the sensors is the output for the wagon.

Furthermore, the maximum lashing force (LF_max_) during transportation is necessary to be measured to help decide the required lashing device.

### Design of simulation conditions

The characteristics of a simulation case contain four elements: the key parameters of the track, the cruise velocity, the location of the cargo, and the stiffness of the lashings.

1. Based on the regulations proposed by the European Committee for Standardization, the processes of curve negotiations will be monitored when the vehicle goes through two tracks, which have a 350 m radius curve and a 600 m radius curve respectively^28,29^. The UIC low defects track irregularity is adopted^30^. The gauge is 1435 mm, and the layouts of two tracks are illustrated in Fig. 5, where *R* (m) denotes the curve radius, *u* (mm) denotes the super elevation.

**Figure 5:**
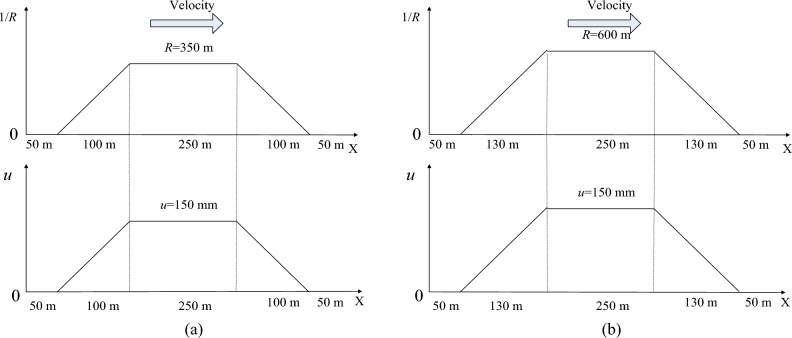
The layout of the tracks: (**a**) The track with a 350 m radius curve; (**b**) The track with a 600 m radius curve.
2. In order to reveal the comprehensive phenomena when the wagon negotiates the curve with different velocities, the simulations should cover the maximum velocity, minimum velocity, and balanced velocity. As Fig. 5 shows, the super elevation has been decided as 150 mm. According to the requirements in EN14363 and EN13803, the maximum cant deficiency and maximum cant excess can be assumed as 130 mm. Then the negotiation velocity can be calculated as
  13 $$ v = \sqrt {\frac{(u + I)Rg}{{1435}}} $$
  where *I* (mm) denotes the cant deficiency and can be replaced by the opposite of cant excess when necessary.
  Based on Eq. (13), the range of test velocities can be obtained. The simulation cases, whose characteristics are made up of the curve radius and cruise velocity, are listed in Table 3.

**Table 3:** The indexes of the simulation cases.

*R* (m)	*v* (m/s)	Case No
350	7	1
350	19 (Balanced velocity)	2
350	26	3
600	9	4
600	25 (Balanced velocity)	5
600	34	6
3. For each simulation case, the wagons with different loading offsets have different running characteristics during curve negotiations. It is assumed that the cargo is loaded evenly in the lateral direction. Longitudinally, the weight of the loaded wagon is distributed to each bogie as Fig. 6 shows, where O denotes the gravity center of the wagon, P denotes the gravity center of the cargo, *x*_*c*_ denotes the longitudinal loading offset, *l* denotes half of the distance between center pivots, *m* denotes the mass of the cargo and it is divided into *m*_*f*_ and *m*_*r*_.

**Figure 6:**
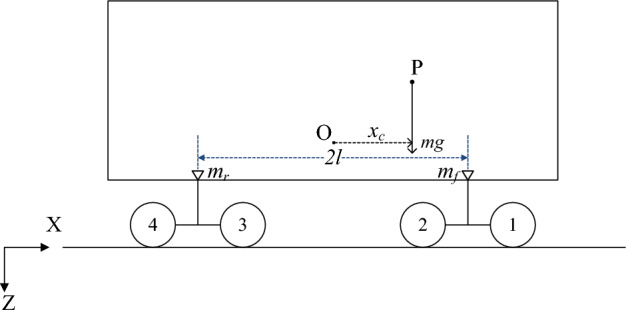
The longitudinal distribution of loading.
  As a basic requirement in the longitudinal direction, the weight of cargo on any truck should not exceed half of the load limit^6,31^, which can be represented by 2*M*. Thus, the maximum loading offset can be obtained as
  14 $$ x_{c\max } = \frac{M \times 2l}{m} - l $$
  Based on the parameters of the wagon, $x_{c\max } \approx 1.8{\text{ m}}$. Thus, the wagons with different values of *x*_*c*_ (1.8/− 1.8/0) are tested in each simulation case.
4. The characteristics of the lashing devices include the pre-tension force and the tensile stiffness. Generally, the pre-tension force is assumed as 500 N for the diagonal lashings^5^. According to the results of current evaluation methods, possible values of the tensile stiffness are designed as (1.2 × 10^7^) N/m, (2.7 × 10^7^) N/m, (4.2 × 10^7^) N/m, (5.7 × 10^7^) N/m, (7.2 × 10^7^) N/m and (8.7 × 10^7^) N/m.

## Numerical verification

In order to examine the accuracy of the wagon model, we adopt the results in the project of “Test of the allowable gravity center height and lateral offset for the loaded wagon with high speed” as reference^32^. In the field test, the vehicle running performances are examined respectively when it negotiates the curves whose radii are 350 m and 600 m. The field test also uses the C_70H_ as the carrier. The characteristics of the loaded wagon in the field test are: (1) the mass of the cargo is 60 tons; (2) *x*_*c*_ = − 0.77 m; (3) the height of the wagon gravity center is 2.3 m.

**Figure 7:**
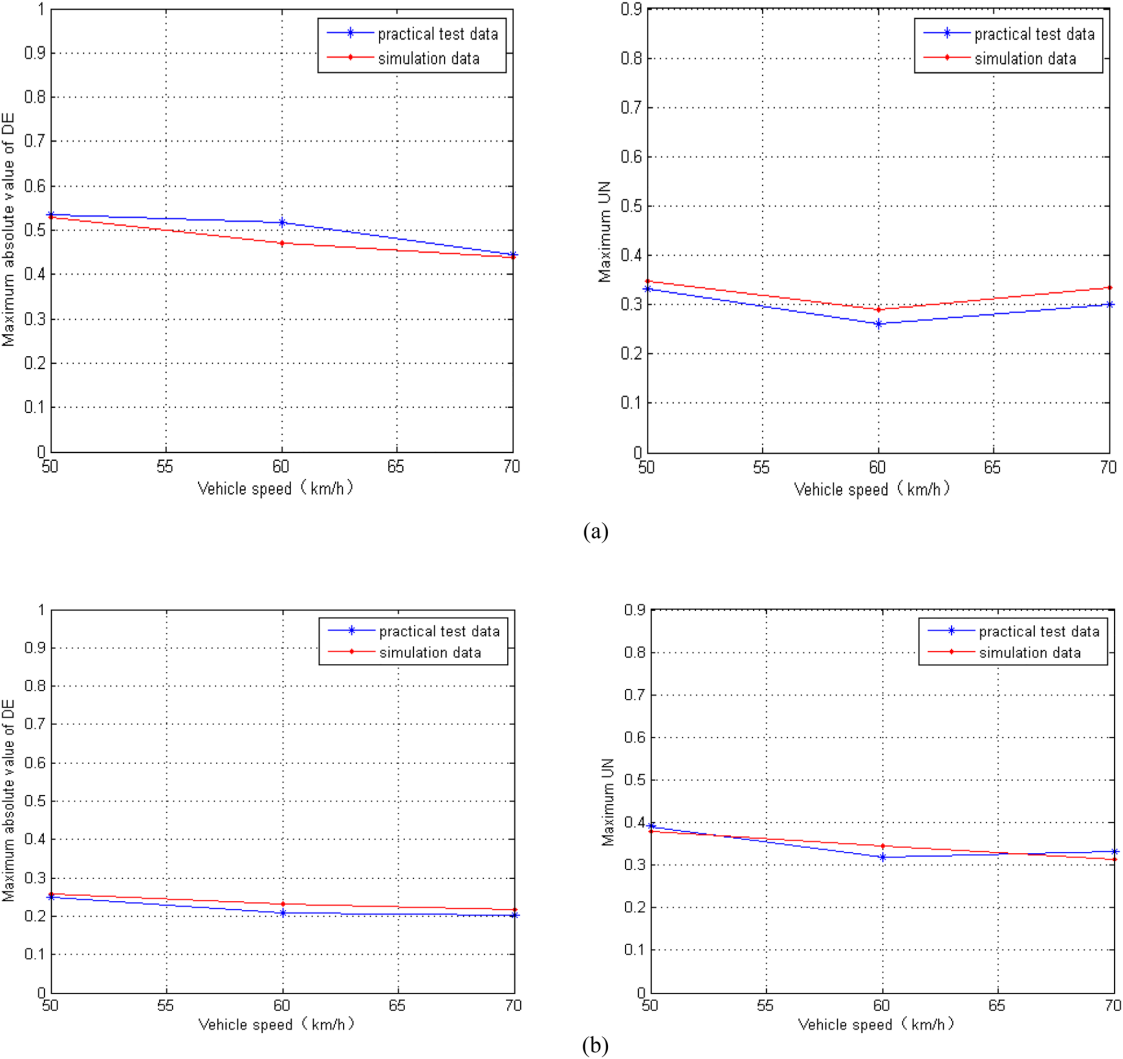
The comparison of different data sources: (**a**) *R* = 350 m; (**b**) *R* = 600 m.

We adjust the parameters of the cargo in the established model to imitate the conditions of the field test. Because the derailment coefficient (DE) and wheel unloading rate (UN) are monitored in the test, we compare the simulation results of DE and UN with the field test results in Fig. 7. We can see that the discrepancies are reasonably within the practical engineering tolerance.

## Simulation results

**Figure 8:**
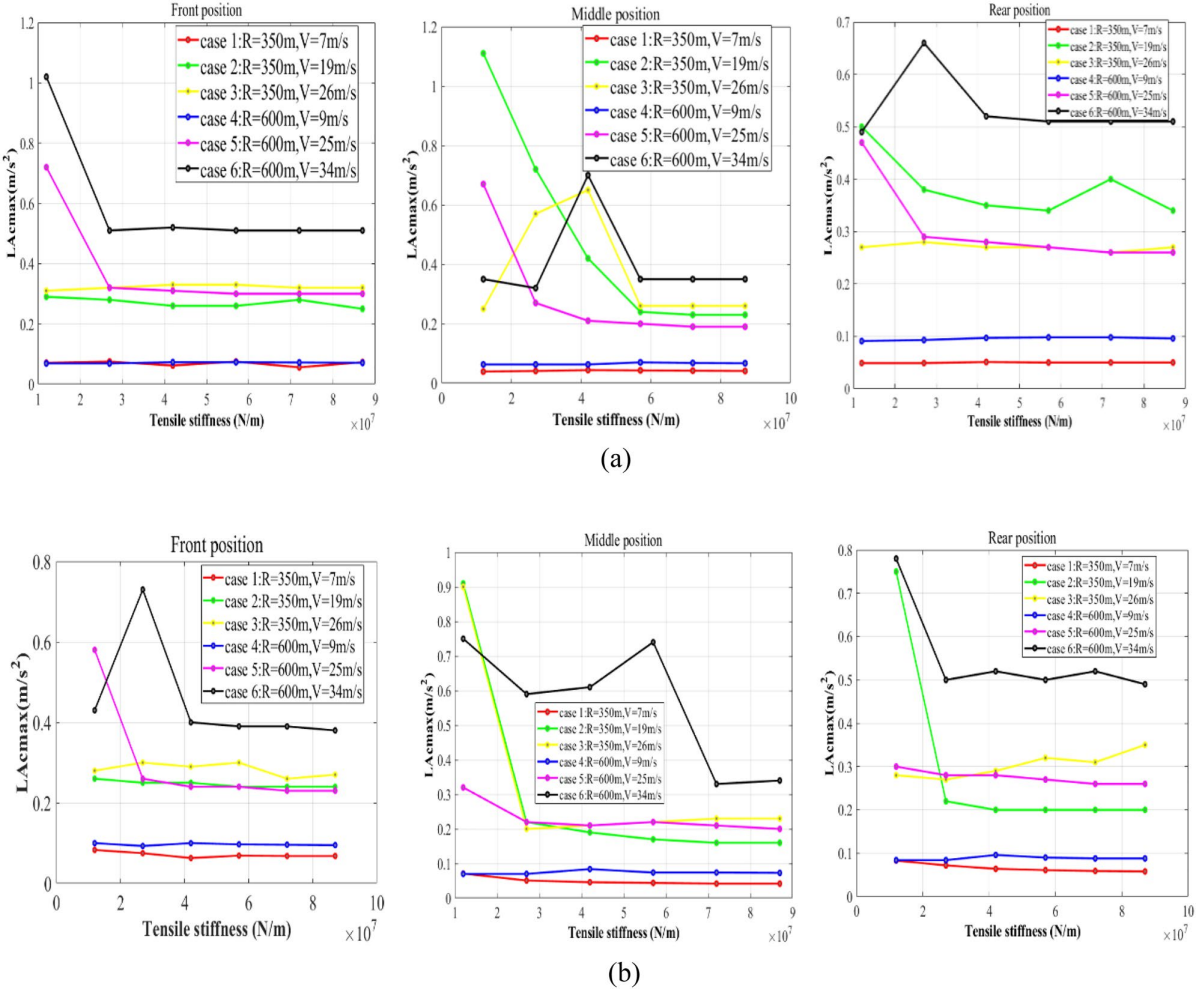
Maximum lateral accelerations of the cargo: (**a**) LA_cmax_ on the circular curve; (**b**) LA_cmax_ on the transition curve.

**Figure 9:**
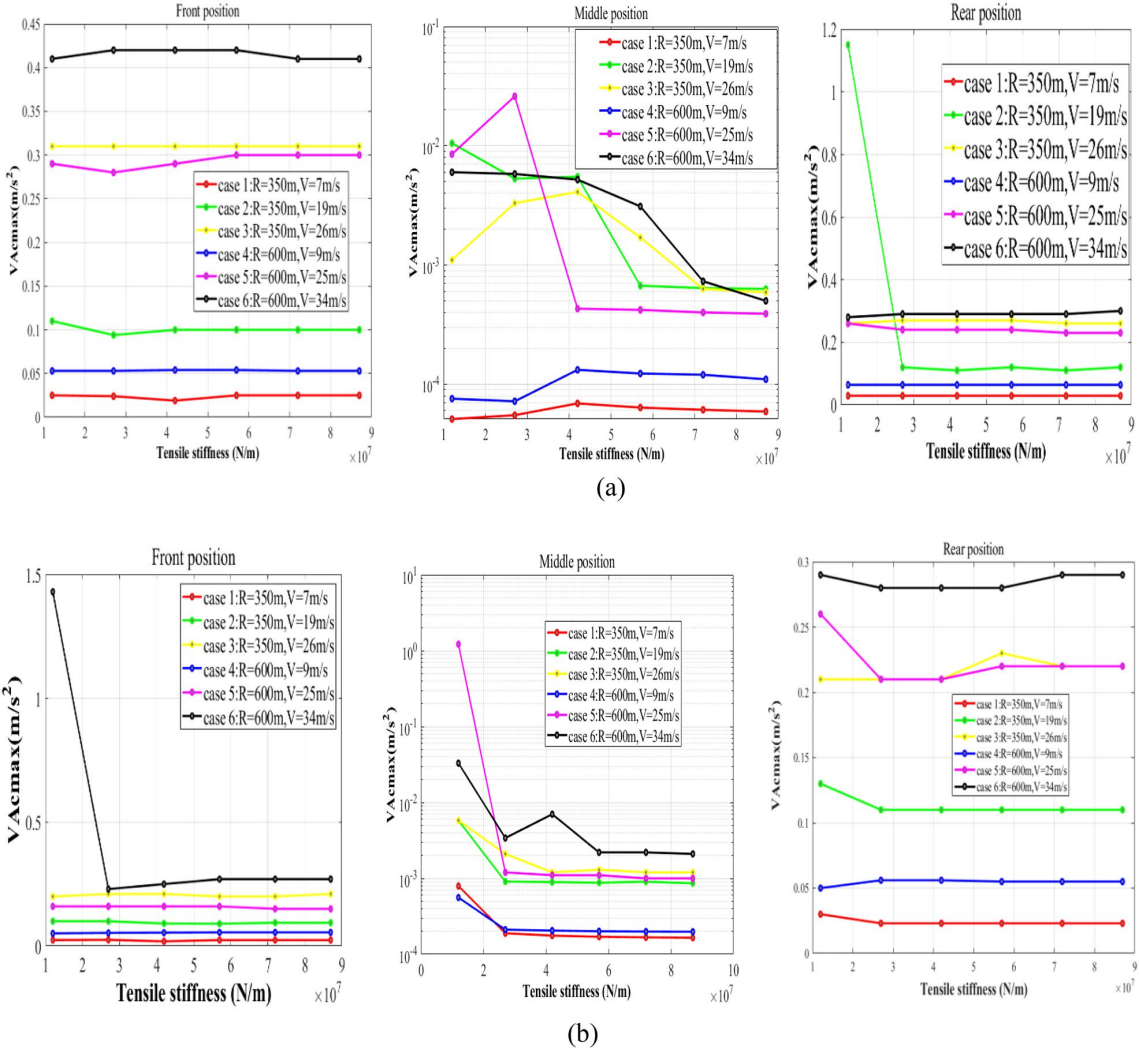
Maximum vertical accelerations of the cargo: (**a**) VA_cmax_ on the circular curve; (**b**) VA_cmax_ on the transition curve.

**Figure 10:**
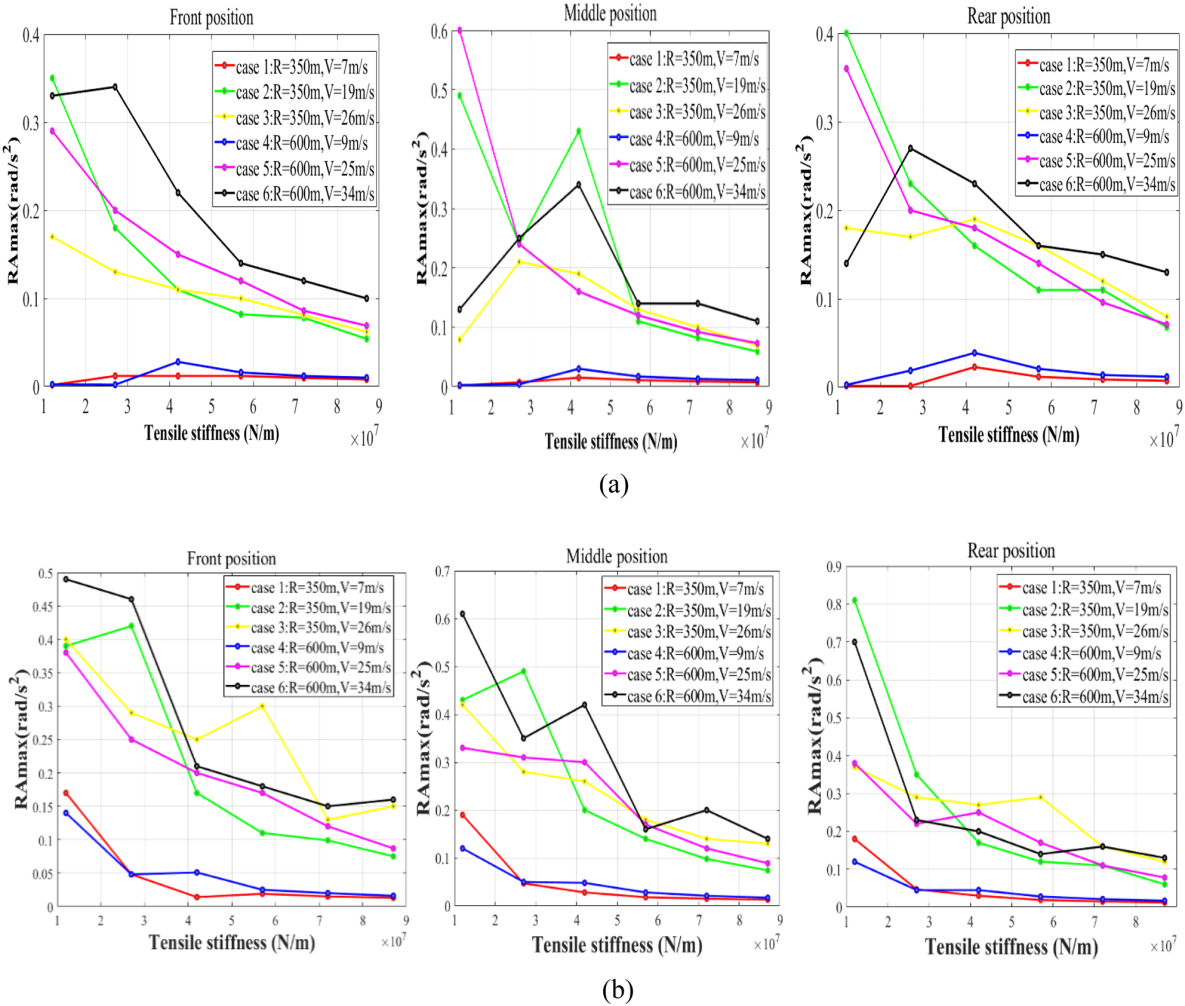
Maximum rolling accelerations of the cargo: (**a**) RA_max_ on the circular curve; (**b**) RA_max_ on the transition curve.

**Figure 11:**
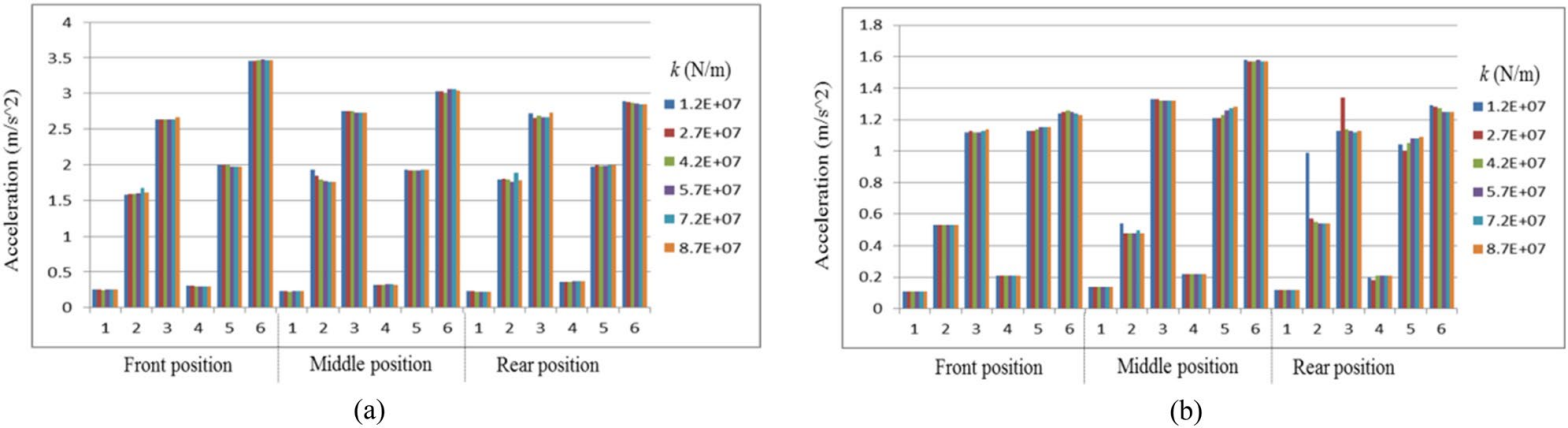
Carbody vibrations for the wagon: (**a**) LA_wmax_; (**b**) VA_wmax_.

**Figure 12:**
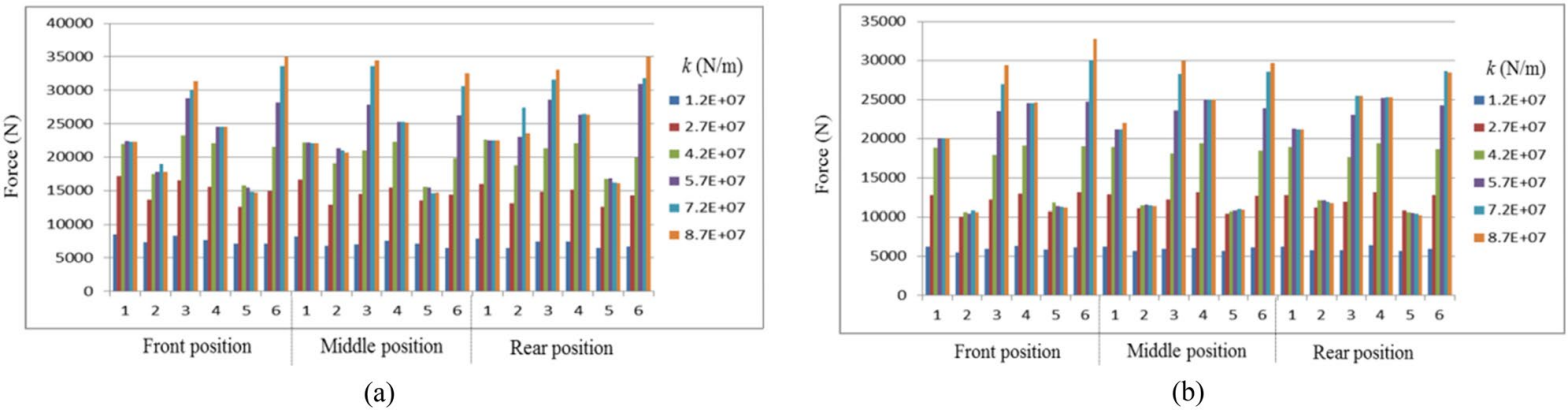
Maximum lashing force: (**a**) LF_max_ on the circular curve; (**b**) LF_max_ on the transition curve.

The comparison of vehicle behaviors between the negotiation process of circular curves and the negotiation process of transition curves is revealed. Different cargo positions on the wagon are considered. In the following figures, “Middle position” means the cargo is evenly located. “Front position” and “Rear position” respectively mean that the cargo is close to the front and rear ends of the carbody with the maximum longitudinal offset. The low pass Butterworth filter with a cut-off frequency of 40 Hz is adopted to deal with the data^33^. For Figs. 8, 9 and 10, the lateral axis denotes the tensile stiffness, and the accelerations are measured at the center of the cargo. For Figs. 11 and 12, the lateral axis denotes the Case number.

### The lateral vibration of the cargo

The maximum lateral accelerations of the cargo (LA_cmax_) in the reference system of the wagon floor are illustrated in Fig. 8.

Three primary conclusions can be drawn based on Fig. 8:

1. Improving the stiffness of lashings can help to suppress the lateral vibration of the cargo.
  Although there is no significant relationship between the stiffness of lashings and the LA_cmax_ when the stiffness of lashings is small, the lateral vibration of the cargo can be controlled at a relatively low level if the stiffness of lashings is large enough.
2. The LA_cmax_ is closely related to the running conditions of the vehicle.
  Case 6 is definitely the most challenging simulation condition. Under most circumstances, the maximum cant deficiency will bring severe lateral vibration of the cargo when the stiffness of lashings is sufficient. Moreover, when the vehicle negotiates curves with the maximum cant excess, the cargo always vibrates gently.
3. The strategy of even-loading can play a role in suppressing the lateral vibration of the cargo on a curve.

### The vertical vibration of the cargo

The maximum vertical accelerations of the cargo (VA_cmax_) in the reference system of the wagon floor are revealed in Fig. 9.

Figure 9 illustrates three effective rules to help suppress the vertical vibration of the cargo:

1. The sufficient stiffness of lashings is necessary for suppressing the vertical vibration of the cargo. If the stiffness of lashings is too small, the cargo may vibrate severely during negotiating the curves.
2. For the uneven-loaded wagon, which should be focused on when studying the vertical vibration of the cargo, the VA_cmax_ is directly affected by the cant deficiency.
3. The VA_cmax_ is significantly affected by the longitudinal loading offset.

It has been proved that the longitudinal loading offset could increase the vertical acceleration of the carbody^34–36^. Obviously, the severe vibration of the carbody will lead to an increase in VA_cmax_. Such regularity is adequately reflected in the simulation results.

### The rotation of the cargo

Apart from the translational vibration, the rotation of the cargo is also a critical threat to the safety of railway freight transportation. The maximum rolling accelerations of the cargo (RA_max_) with respect to the reference base of the wagon floor are illustrated in Fig. 10.

Figure 10 gives us full evidence to point out that:

1. The increase in the lashing stiffness plays an important role in suppressing the rotation of the cargo.
2. As for the wagon with sufficient lashing stiffness, the RA_max_ increases with the rise in the cant deficiency and velocity. When the wagon negotiates curves with the maximum cant excess, the rotation of the cargo can remain slight.
3. The rotation of the cargo has no obvious relationship with the loading offset.

### The vibration of the carbody

The major goal of the investigation into the vibration of the carbody is to reveal the effects of load restraint assemblies and vehicle running conditions on the stability of the vehicle during the curve negotiation. For the sake of avoiding redundant statements, we only display the simulation results during negotiating the circular curve, considering the similar characteristics between the processes of negotiating the circular curve and the transition curve. The maximum lateral and vertical accelerations of the carbody (represented by LA_wmax_ and VA_wmax_ respectively) are revealed in Fig. 11.

We can draw the conclusions as below:

1. If the stiffness of lashings was too small, the stability of the wagon would be threatened.
2. Basically, the vibration of the carbody is closely affected by the running conditions. It should be noted that there is a small difference between the features of LA_wmax_ and VA_wmax._ The LA_wmax_ has a greater relationship with the cant deficiency. Comparatively speaking, the VA_wmax_ has a greater relationship with the cruise velocity.
3. The loading offset has no significant effect on the stability of the wagon.

### The maximum lashing force during the transportation

As for the established model, four lashings offer the constraining force. The maximum lashing force (LF_max_) for any lashing is illustrated in Fig. 12.

Figure 12 reveals the characteristics of the LF_max_ as below:

1. As the lashing stiffness increases, the LF_max_ will gradually increase until the lashing stiffness is large enough. At this “Growth Stagnation” stage, the LF_max_ will slow down, keep steady, or even decrease.
2. Considering the phenomenon during the “Growth Stagnation” stage, there is a higher requirement on the lashing capacity when the wagon negotiates curves with the maximum cant deficiency.

## Discussion

In Section “Simulation results”, we reveal the simulation results of all the assessment indices, which show that the stability of the cargo will be improved with the increase of the lashing stiffness. This is an important finding in the evaluation of the load restraint assemblies. Because of the lack of limit values, we decide to confirm the necessary lashing stiffness as (7.2 × 10^7^) N/m based on the tendency of all the indices, no matter what the longitudinal offset is. Then according to Fig. 12, the minimum lashing capacity can be obtained. As a comparison, the decisions on the lashing devices are listed in Table 4, where *x*_*c*_ denotes the longitudinal loading offset, *k* denotes the lashing stiffness and *LC*_min_ denotes the necessary lashing capacity.

**Table 4:** The calculation results of different methodologies.

*x*_*c*_ (m)	Method	*k* (N/m)	*LC*_min_ (kN)
0	Method of CR	1.20 × 10^7^	65
	Method of CEN	2.71 × 10^7^	139
	Dynamics simulation	7.20 × 10^7^	34
1.8	Method of CR	2.71 × 10^7^	136
	Method of CEN	2.71 × 10^7^	139
	Dynamics simulation	7.20 × 10^7^	34
− 1.8	Method of CR	2.71 × 10^7^	136
	Method of CEN	2.71 × 10^7^	139
	Dynamics simulation	7.20 × 10^7^	32

It is worth discussing these interesting facts revealed by the comparison in Table 4. On one hand, the results of the dynamics simulation now provide evidence to weaken the role of *x*_*c*_ in the evaluation of load restraint assemblies. On the other hand, superior results are achieved with the method of the dynamics simulation considering the values of assessment indices shown in Section “Simulation results”. Thus, we hold that it is necessary to adopt the method of dynamics simulation due to its consideration of the relative motion between the cargo and the wagon floor.

## Conclusions

Because of the super elevation and the centrifugal force, the vibration of goods during the curve negotiation is a serious threat to the safety of railway freight transportation. This paper proposes an effective method of dynamics simulation to evaluate the necessary load restraint assemblies to suppress cargo vibration.

Two traditional methods are used to evaluate the load restraint assemblies. Then, based on the dynamics function of the cargo and the existing knowledge about the vehicle dynamics, the simulation model is established and validated by the field test data. Based on the designed assessment indices and the simulation conditions, the wagon-cargo coupled model is simulated under different running and loading conditions. The numerical studies demonstrate that the lashing stiffness plays an important role in the stability of the cargo, as well as the maximum lashing force. Comparatively speaking, the stability of the wagon is more related to vehicle running conditions. Based on the simulation results, the characteristics of lashing devices are decided. The superiority of the proposed method is proven.

We hope the proposed methodology and conclusions in this paper can cast a new light on the evaluation of load restraint assemblies.

## Data Availability

All data generated or analysed during this study are included in this published article.
